# 629. Investigating the Molecular Epidemiology of Extended-Spectrum β-Lactamase Producing Enterobacterales Among Patients Admitted to the Intensive Care Unit

**DOI:** 10.1093/ofid/ofaf695.196

**Published:** 2026-01-11

**Authors:** Sima L Sharara, Patricia Simner, Yehudit Bergman, Emily B Jacobs, Suiyini Fiawoo, Eili Klein, Sara E Cosgrove, Pranita Tamma

**Affiliations:** Johns Hopkins Hospital, Baltimore, MD; Mayo Clinic, Rochester, Minnesota; Johns Hopkins, Baltimore, Maryland; Johns Hopkins School of Medicine, Timonium, Maryland; Johns Hopkins School of Medicine, Timonium, Maryland; Johns Hopkins School of Medicine, Timonium, Maryland; Johns Hopkins School of Medicine, Timonium, Maryland; Johns Hopkins University School of Medicine, Baltimore, Maryland

## Abstract

**Background:**

Colonization with extended-spectrum β-lactamase-producing Enterobacterales (ESBL-E) is increasingly recognized in critically ill patients and impacts infection prevention practices and empiric antibiotic selection. However, contemporary United States estimates of ESBL-E prevalence at the time of ICU admission are limited.

**Table 1.**
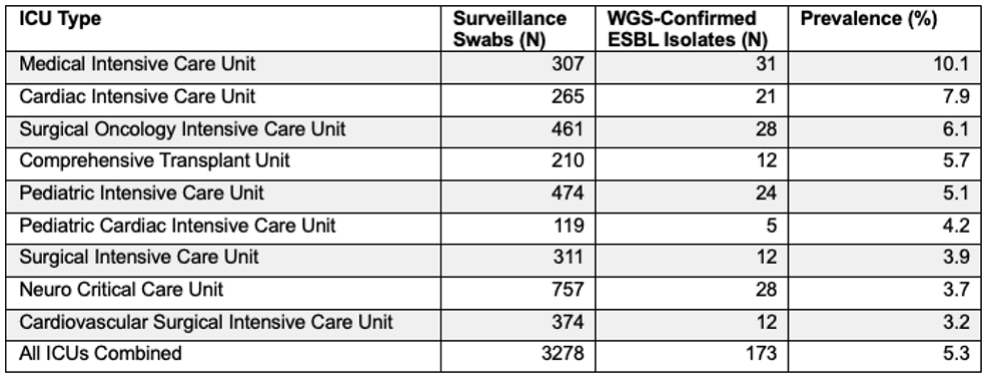
ICU-Specific Prevalence of ESBL Colonization at Admission Confirmed by Whole Genome Sequencing

**Figure 1.**
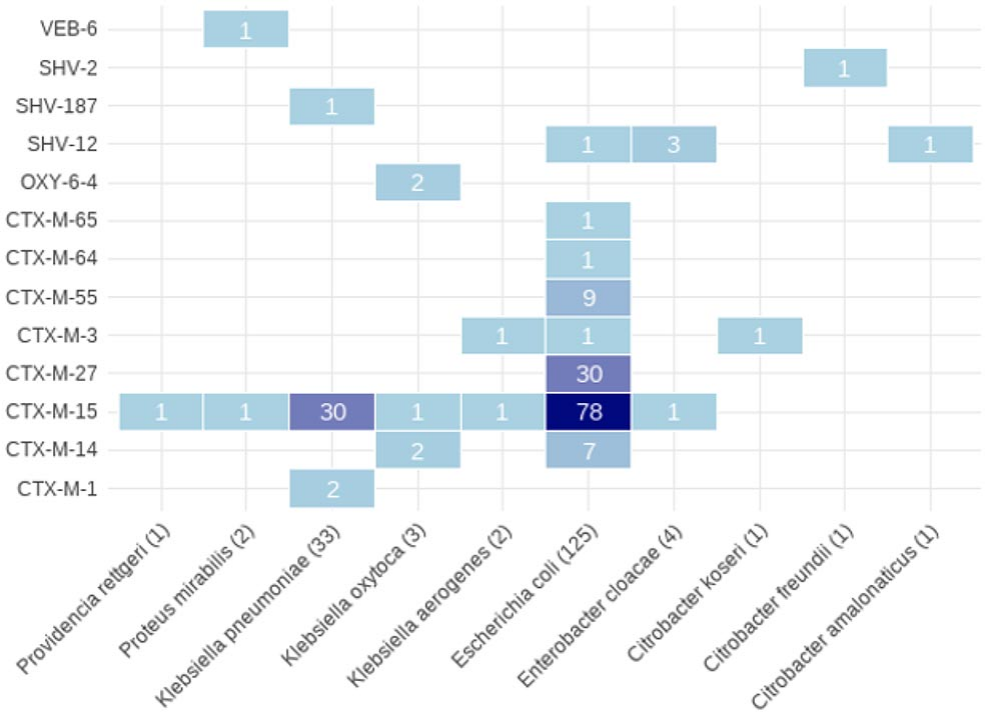
Distribution of ESBL Gene Variants Among ESBL-Producing Enterobacterales Isolates by Bacterial Species (n = 173)

**Methods:**

We conducted a prospective molecular epidemiology study of ESBL-E colonization among adult and pediatric patients admitted to nine ICUs at The Johns Hopkins Hospital between March and August 2023. Routine rectal surveillance swabs (Copan Eswab) collected at ICU admission were plated on selective chromogenic media (HardyCHROM™ ESBL) to identify third-generation cephalosporin-resistant Enterobacterales (3GCRE). Species identification was performed using MALDI-TOF MS, and ceftriaxone resistance was confirmed by broth microdilution. Whole-genome sequencing (WGS) was performed on the first isolate of each species per patient to confirm ESBL gene carriage and assess molecular features.

**Results:**

Among 3,278 ICU admissions, 328 (10%) had 3GCRE isolated on admission swabs. Of these isolates, 173 (5% of all admissions) were confirmed by WGS to harbor at least one ESBL gene—representing approximately 53% of all 3GCRE isolates. ESBL-E colonization was most prevalent in the medical (10%) and cardiac (8%) ICUs. The most common ESBL-E species were *Escherichia coli* (72%) and *Klebsiella pneumoniae* (19%). Dominant *E. coli* sequence types were ST131 (36%), ST1193 (7%), and ST38 (6%). Nearly all ESBL genes belonged to the *bla*_CTX-M_ family (95%), with *bla*_CTX-M-15_ as the predominant allele (63%). Other ESBL genes included *bla*_CTX-M-27_ (17%), *bla*_CTX-M-14_ (5%), *bla*_CTX-M-55_ (5%), and *bla*_SHV-12_ (3%).

**Conclusion:**

In this large, prospective cohort, 5% of ICU patients were colonized with ESBL-E at admission, most commonly *E. coli* and *K. pneumoniae* harboring *bla*_CTX-M_ genes. Phenotypic detection of 3GCRE overestimated ESBL-E prevalence by nearly 90%, highlighting the need for molecular confirmation to accurately characterize resistance. These findings suggest ESBL-E colonization remains uncommon in the U.S., even at a major referral center, supporting non-carbapenem empiric therapy in low-risk patients and reinforcing the need for targeted surveillance.

**Disclosures:**

All Authors: No reported disclosures

